# Poor in vitro fertilisation outcomes in genital tuberculosis – Case report

**DOI:** 10.1016/j.amsu.2022.104438

**Published:** 2022-08-19

**Authors:** Dian Tjahyadi, Kevin Dominique Tjandraprawira

**Affiliations:** a Department of Obstetrics and Gynaecology, Faculty of Medicine, Dr. Hasan Sadikin General Hospital, Universitas Padjadjaran, Bandung, Indonesia; b Bandung Fertility Center, Limijati Women and Children Hospital, Bandung, Indonesia

**Keywords:** Genital tuberculosis, Assisted reproductive technology, In vitro fertilisation

## Abstract

**Introduction and importance:**

Genital tuberculosis (TB) exerts significant damage in the reproductive organs, particularly the Fallopian tubes and endometrium. Infertility is one of the most common presenting causes, often subsequently requiring assisted reproductive technology (ART). However, we have not had many experiences with genital TB despite being a country endemic for TB. This case series highlights the challenges we face and the solutions we wish we had.

**Case presentation:**

In this case series, we recruited 7 patients undergoing *in vitro fertilisation* (IVF) treatment previously diagnosed with TB between January 01, 2014 and June 30, 2021. Patients were recruited at the beginning of their IVF treatments. Of 7 patients, 2 patients (28.6%) achieved live birth. 5 patients (71.4%) failed to conceive. All patients had good and/or excellent quality embryos upon transfer but only 2/7 managed to conceive and delivered.

**Clinical discussion:**

Genital TB is often silent and only encountered during workup for infertility. Genital TB often produces extensive damage on the linings of the endometrium and Fallopian tubes, accounting for the recurrent implantation failures associated with the disease. Whilst antitubercular treatment may improve the prognosis, many women still fail to conceive.

**Conclusion:**

Genital tuberculosis remains a significant issue in infertility. Cases are often silent and management is often delayed. IVF is often required due to the longstanding damage caused beforehand yet prognosis may remain poor.

## Highlights

• Genital tuberculosis is a significant yet underdiagnosed etiology of infertility.
• Its profound severity often requires *in vitro* fertilisation for conception.
• Poor prognosis may still be found despite IVF due to recurrent implantation failures.

## 1 Introduction

Genital tuberculosis (TB) is a rare complication of TB [1]. It spreads predominantly hematogenously from a primary site of infection in the lungs [2]. Genital TB is known to predominate in specific areas of the reproductive organs, in particular the Fallopian tube and the endometrium [2]. Genital TB produces extensive damage in the reproductive organs, disrupting the fine villial lining of the Fallopian tube and decimating the ever-renewing endometrium through the formation of granulomas and caseous necrosis [1]. Due to the extensive damage caused, patients often present with infertility and their diagnoses confirmed upon laparoscopy [1]. Their infertility diagnosis often conveys the need to resort to *in vitro* fertilisation (IVF) for their conception [2].

However, the outcomes have not been extensively described and explored, in particular due to the low and rare cases of genital TB and also the even fewer of such women resorting to IVF for treatment. As a country endemic for TB, this is our opportunity to describe how IVF may be used for overcoming infertility, the challenges present and the solutions we wish we had.

## 2 Case description

We recruited 7 patients who underwent IVF treatment in our facility having been previously diagnosed with TB infection in their reproductive organs between January 1, 2014 and June 30, 2021. The patients were recruited at the beginning of their IVF treatments and were followed up until the completion of their treatment and/or their pregnancies. The cases are reported in line with the SCARE 2020 Guideline [3]. The study's registration on Clinicaltrials.gov is NCT05428124 (https://clinicaltrials.gov/ct2/show/NCT05428124).

### 2.1 Case 1

A 31-year old P0A0 presented with primary infertility. She had been married for 8 years. She was diagnosed with tuberculous salpingitis after laparoscopy. She had had a history of tuberculous lymphadenitis in 2006 for which she underwent complete treatment. Her husband was normozoospermic. Her AMH level was 1.5 ng/mL.

She commenced her IVF treatment on April 22, 2014. She was prescribed 225 IU of FSH injections for her ovulation. She had 8 oocytes retrieved on May 5, 2014.5 oocytes were fertilized, of which 2 were excellent and 3 were good. All of them were frozen.

She underwent a frozen embryo transfer on June 29, 2014 during which 2 excellent and 1 good embryos were transferred.

She successfully conceived and would later gave birth to a healthy female term newborn.

### 2.2 Case 2

A 33-year old P0A0 came to our facility with primary infertility, having been married for 8 years. She had undergone a laparoscopy during which she was diagnosed with tuberculous salpingitis. She had had a history of pulmonary TB the previous year and had undergone treatment for her TB. Her husband was asthenoteratozoospermic. Her AMH level was 2.71 ng/mL.

She commenced her IVF treatment on July 14, 2020 with 225 IU of FSH injections. She had her oocytes retrieved on July 26, 2020 during which 21 oocytes were retrieved and they were all frozen.

On October 26, 2020, 2 embryos (both blastocysts, of excellent quality) were transferred. She still has 4 remaining embryos in storage. She was confirmed to be pregnant soon after and on June 17, 2021, gave birth to a healthy boy, 3900 g, 49 cm through caesarean section. She made an uneventful recovery prior to being discharged a few days after.

### 2.3 Case 3

A 30 y.o P0A1 with secondary infertility presented to our facility. She had been married for 3 years. She had undergone a laparoscopy about a year prior to presentation and she was diagnosed with bilateral chronic tuberculous salpingitis. Her husband was normozoospermic. Her AMH level was 1.90 ng/mL.

She underwent IVF with a long protocol on September 3, 2019. She was prescribed on 150 IU of FSH injections to trigger her ovulation. She had her oocytes retrieved on September 30, 2019 during which 8 oocytes were collected. 3 oocytes were fertilized and they were transferred as day 3 embryos (all of good quality). Unfortunately, she did not become pregnant and had her menstruation on October 21, 2019.

### 2.4 Case 4

A 41 y.o P0A1 with primary infertility presented to our facility. She had been married 14 years and had a history of primary pulmonary TB when she was 16 years old after which she underwent complete treatment. She underwent a laparoscopy surgery for bilateral tubal obstructions in 2015. However, she underwent another laparoscopy in 2018 during which she was discovered to have genital tuberculosis. Her husband was normozoospermic and her AMH level was 1.67 ng/mL.

She had had 4 previous rounds of IVF and 2 failed frozen embryo transfers prior to presenting to our facility. She was recommended to undergo another IVF cycle with the short protocol which commenced on March 4, 2021. She was given 75 IU of FSH injections to trigger her ovulation.

Oocyte retrieval was on March 16, 2021 during which 9 oocytes were retrieved. 3 fertilized oocytes were produced and they were transferred as day 3 embryos (2 were excellent, 1 was good) on March 19, 2021. Unfortunately, she failed to conceive again and had her menstruation on April 4, 2021.

### 2.5 Case 5

A 27-year old P0A0 having been married for 1 year, presented to our facility with primarily infertility. She had undergone a laparoscopy during which the presence of tuberculous salpingitis was confirmed on both her Fallopian tubes. Her husband was teratoospermic. Her AMH level was 1.45 ng/mL.

She started her IVF treatment with a short protocol on January 27, 2021. She was prescribed 75 IU of FSH injections to trigger her ovulation. She had her oocytes retrieved on February 8, 2021 during which 4 oocytes were retrieved. Only one good embryo was produced and it was transferred on February 11, 2021.

Unfortunately, she failed to conceive.

### 2.6 Case 6

A 28-year old P0A0 presented to our facility with primary infertility. She had been married for 4 years. She had previously undergone laparoscopy during which she was discovered to have tuberculosis on her right ovary and Fallopian tube and left Fallopian tube obstruction. Her husband was normozoospermic. Her AMH level was 1.56 ng/mL.

She was prescribed to undergo IVF with a long protocol on August 7, 2019. She was prescribed on 75 IU of FSH injections to trigger her ovulation. On September 2, 2019, she had 12 oocytes retrieved from her follicles.

She had 2 embryos transferred (both blastocysts, 1 excellent and 1 good) on September 7, 2019. She did not have any embryos in storage. Unfortunately, she failed to conceive after her embryo transfer.

### 2.7 Case 7

A 30-year old P0A0 presented with primary infertility to our facility. She had been married for around 6 years. She had had a laparoscopy 1 year prior during which bilateral hydrosalpinges were discovered and confirmed tuberculous salpingitis. Her husband was teratozoospermic. Her AMH level as 1.63 ng/mL.

She was then recommended to undergo IVF with a long protocol, starting on June 20, 2020. She was prescribed on 150 IU of FSH injections to trigger her ovulation. On June 13, 2020, she underwent oocyte retrieval during which 7 oocytes were retrieved.

6 fertilized eggs were produced of which 3 excellent grade embryos were transferred on June 16, 2020.3 other embryos (2 excellent grade and 1 good grade) were cryopreserved. Unfortunately, she failed to conceive.

## 3 Discussion

Tuberculosis is endemic in Indonesia and Indonesia is ranked 2nd after India in the world for TB prevalence [4]. Tuberculosis causes widespread systemic manifestations, including genital TB [4]. Female genital TB is often a secondary complication of pulmonary TB or primary TB located outside the genital system [1,4]. It is estimated that around 8–15% of pulmonary TB patients will have a manifestation in the genital organs, even though they are often asymptomatic [1]. Unfortunately, due to the silent nature of genital TB infections, there is neither specific data nor official reports about genital TB in Indonesia [4].

After initial pulmonary infection, the mycobacteria are spread hematogenously or lymphatically and they would then have a preference towards invading the Fallopian tubes [1,2]. The Fallopian tubes are almost always involved and they are often bilaterally involved [1]. Once an infection is established in the endosalpinx, it gradually spreads to the endometrium, the ovaries and occasionally to the cervix and would rarely to the vagina [1,2]. Direct spread to the Fallopian tubes is established as tubercles on the surface [2]. Interestingly, the myometrium appears to be more resistant towards TB [1]. When the primary TB focus is in the abdomen or the peritoneum, it might be a direct spread to the genital system [1,2].

It is notable that among our patients, only 2 patients (28.6%) had a confirmed history of pulmonary TB. They were both treated for their previous TB infections but even adequate treatment still rendered these patients at risk for extrapulmonary TB infections. The pathophysiology of TB infections indicates that primary point of infection would have always been the lungs. The silent nature of pulmonary TB in these cases, which led to extrapulmonary manifestations, has made TB workup a lot more difficult.

Female genital TB is most often silent and asymptomatic [1]. The lack of diagnostic means for genital TB means it is often only stumbled during laparoscopic surgeries as part of an infertility workup [4]. Specific surgical findings such as adhesions, necrotic tissues coupled with tubal obstruction are common. History of pulmonary and/or extrapulmonary TB is often found but not necessarily pathognomonic. As a result, patients are often late in their presentation [4]. Infertility remains the most common complaint and it is attributed to the tubal damage and adhesions in various areas of the genital organs due to the chronic inflammatory course [1,4]. Tubal obstruction may form hydrosalpinges or pyosalpinges [2]. Ovum transport is impeded and the uterine cavity may be distorted due to adhesions and synechiae [1,2]. Genital TB may also lead to ovarian damage which leads to a significant reduction in ovarian reserve [5]. This is common in latent genital tuberculosis even though the mechanism remains unclear [5].

Menstrual disorders are also present in pulmonary TB without apparent genital involvement [1]. In cases of genital tuberculosis, endometrial involvement is often the etiology of menstrual disorders [1]. The disorders may include amenorrhea, dysmenorrhea, oligohypomenorrhea, menorrhagia, menometrorrhagia and postmenopausal bleeding [1].

Endometrial metabolism abnormalities are noted in genital tuberculosis, including in dormant female genital tuberculosis [6]. Subramani et al. noted that significant endometrial tissue metabolites occur, largely related to energy metabolism and amino acid biosynthesis [6]. There was increased production of various amino acids in women with dormant genital TB coupled with a significant decrease in glucose production [6]. Such abnormalities contribute to implantation failures common in genital TB [6].

Once genital TB is diagnosed, antituberculosis treatment is imperative. Treatment for uncomplicated genital TB lasts for 6 months, initiated by giving daily therapy of rifampicin, isoniazid, pyrazinamide and ethambutol for 2 months followed by 4 months of daily therapy of rifampicin and isoniazid [7]. Complicated cases, such as among those relapsing and/or with immunosuppressive conditions would require therapy for 9–12 months.

Treatment for endometrial tuberculosis is known to improve fertility prognosis. Jindal et al. discovered that amongst women with silent endometrial tuberculosis, the administration of anti-tubercular treatment (ATT) improved the chances of spontaneous conception [8]. They discovered that >90% of women would conceive within the first 12 months, i.e. during the period of ATT administration or within 6 months of treatment completion, compared to women with unexplained infertility lacking mycobacterial involvement [8]. Whilst their study involved women with only PCR results positive for *Mycobacterium tuberculosis* as the sole indication of endometrial tuberculosis, their study has renewed hope that amongst women with evident genital tuberculosis, ATT administration should improve fertility outcomes [8]. Still, the results of their study should be interpreted with caution. Their study implied that the endometrial damage may not have been extensive and that the presence of mycobacteria had not produced extensive damage in the tubes. In cases where damage is ‘too far gone’, spontaneous conception will not be possible and assisted reproductive technologies should be sought.

Genital tuberculosis also impairs ovarian reserve. Malhotra et al. reported that women with genital TB undergoing IVF in India show significantly higher mean FSH and LH levels with significantly lower inhibin B levels when compared to controls [9]. Stromal vasculature correlates with ovarian reserve as optimal blood supply would ensure proper developments of follicles in response to high doses of exogenous gonadotropins [9]. It has been reported that stromal blood flow in the ovaries is compromised in genital TB [9]. It is suspected that TB infection involving the ovaries would form fibrosis, causing periovarian adhesions [9]. These adhesions may then compromise ovarian reserve by restricting ovarian blood supply [9].

Due to the widespread damage caused, conception is often possible only through assisted reproductive technologies (ART) [10]. When tubal and/or endometrial damages preclude spontaneous conception, IVF and/or intracytoplasmic sperm injection (ICSI) may be the only option available for couples [11]. The use of IVF has long been recommended for genital TB [11]. However, IVF success is related to the site of damage caused by genital TB [12]. Dai et al. conclusively pointed out that the prognosis is poorer when extensive endometrial damage is present compared to when there is only extensive tubal damage [12]. They demonstrated that patients with endometrial tuberculosis had significantly reduced endometrial thickness, high-quality embryo rates and implantation rates when compared with controls [12]. This led to the significantly lower cumulative pregnancy rates of women with endometrial tuberculosis [12]. However, IVF/ICSI pregnancy outcomes showed no significant difference among patients with tubal tuberculosis when compared to controls [12].

However, even with IVF repeated implantation failures is often recorded [10]. Mycobacteria present in the basal endometrial layer may reduce subendometrial blood flow and interfere with implantation [10]. Altered immune response in tuberculosis is known to activate antiphospholipids, which in turn activates antiphospholipid antibodies and microthrombosis, which leads to implantation failures [10].

Our cases all involved tubal disease due to *Mycobacterium tuberculosis*. Most often the tubal obstruction was bilateral, which naturally precluded the ability to spontaneously conceive. It was logical to resort to IVF/ICSI to attempt for conception. However, in our cases, only 2 out of 7 patients (28.6%) managed to conceive and we suspected that the failures were all due to implantation failures. Since genital tuberculosis would often involve not just the Fallopian tubes, it would be logical to assume that for those who failed to conceive, their endometrium might have been impaired as well. Even if the endometrial lines appeared thick enough on ultrasound, it might be assumed that the endometrial blood flow could have been impaired by endometrial manifestations of TB infections. To confirm such hypothesis, it would have been necessary to perform endometrial biopsy on our patients failing to conceive. Unfortunately, we could not perform such procedure due to the hesitancy of our patients for further invasive management following failed IVF cycles.

Our case series is not sufficient to produce any conclusive remarks on the best management of genital TB-related infertility. Our facility is one of the largest fertility centers in our province yet our experience with genital TB-related infertility remains limited. This highlights the difficulties such cases present and the lack of awareness still prevalent today. Systematic and large-scale observational studies would be required to explore the best approach for genital TB-related infertility.

To conclude, genital TB remains a complicated etiology of female infertility. Its widespread damage often precludes any chances of spontaneous conceptions. Unfortunately, it may also limit the chances of IVF/ICSI success when extensive endometrial involvement is present. Prognosis for genital TB-related infertility is unlikely to be improved significantly in the near future.

## Ethical approval

The institutional review board has determined that our study is exempt from ethical approval as it is a review.

## Please state any sources of funding for your research

The authors declared that we did not receive any external funding for our study.

## Author contribution

DT and KDT conceived the study. DT recruited and examined the patients. DT and KDT drafted the manuscript. Both authors agreed on this final version to be published.

## Please state any conflicts of interest

The authors declare that we do not have any conflicts of interests.

## Consent

Written informed consent was obtained from the patients for publication of this case report. A copy of their written consents is available for review by the Editor-in-Chief of this journal upon written request.

## Data availability statement

Anonymised patient data are available upon reasonable written request.

## Permission to reproduce material from other sources

We do not reproduce materials from other sources for this article.

## Provenance and peer review

Not commissioned, externally peer-reviewed.

## Registration of research studies

The study's registration on Clinicaltrials.gov is NCT05428124 (https://clinicaltrials.gov/ct2/show/NCT05428124).

## Guarantor

Dian Tjahyadi as the first author is the guarantor of this study.

## References

[1] Neonakis I.K., Spandidos D.A., Petinaki E. (2011). Female genital tuberculosis: a review. Scand. J. Infect. Dis..

[2] Gatongi D.K., Gitau G., Kay V., Ngwenya S., Lafong C., Hasan A. (2005). Female genital tuberculosis. Obstet. Gynaecol..

[3] Agha R.A., Franchi T., Sohrabi C., Mathew G., Kerwan A. (2020). The SCARE 2020 guideline: updating consensus surgical CAse REport (SCARE) guidelines. Int. J. Surg..

[4] Djuwantono T., Permadi W., Septiani L., Faried A., Halim D., Parwati I. (2017). Female genital tuberculosis and infertility: serial cases report in Bandung, Indonesia and literature review. BMC Res. Notes.

[5] Jirge P.R., Chougule S.M., Keni A., Kumar S., Modi D. (2018). Latent genital tuberculosis adversely affects the ovarian reserve in infertile women. Hum. Reprod..

[6] Subramani E., Jothiramajayam M., Dutta M., Chakravorty D., Joshi M., Srivastava S. (2016). NMR-based metabonomics for understanding the influence of dormant female genital tuberculosis on metabolism of the human endometrium. Hum. Reprod..

[7] Sharma J.B. (2015). Current diagnosis and management of female genital tuberculosis. J. Obstet. Gynaecol. India.

[8] Jindal U.N., Verma S., Bala Y. (2012). Favorable infertility outcomes following anti-tubercular treatment prescribed on the sole basis of a positive polymerase chain reaction test for endometrial tuberculosis. Hum. Reprod..

[9] Malhotra N., Sharma V., Bahadur A., Sharma J.B., Roy K.K., Kumar S. (2012). The effect of tuberculosis on ovarian reserve among women undergoing IVF in India. Int. J. Gynecol. Obstet..

[10] Dam P., Shirazee H.H., Goswami S.K., Ghosh S., Ganesh A., Chaudhury K. (2006). Role of latent genital tuberculosis in repeated IVF failure in the Indian clinical setting. Gynecol. Obstet. Invest..

[11] Soussis I., Trew G., Matalliotakis I., Margara R., Winston R.M. (1998). In vitro fertilization treatment in genital tuberculosis. J. Assist. Reprod. Genet..

[12] Dai W., Ma L., Cao Y., Wu D., Yu T., Zhai J. (2020). In vitro fertilization outcome in women with endometrial tuberculosis and tubal tuberculosis. Gynecol. Endocrinol..

